# Oncogenesis, Microenvironment Modulation and Clinical Potentiality of FAP in Glioblastoma: Lessons Learned from Other Solid Tumors

**DOI:** 10.3390/cells10051142

**Published:** 2021-05-10

**Authors:** Yixin Shi, Ziren Kong, Penghao Liu, Guozhu Hou, Jiaming Wu, Wenbin Ma, Xin Cheng, Yu Wang

**Affiliations:** 1 Department of Nuclear Medicine, Peking Union Medical College Hospital, Chinese Academy of Medical Sciences and Peking Union Medical College, Beijing 100730, China; shiyx16@mails.tsinghua.edu.cn (Y.S.); 15611145656@163.com (G.H.); 2 Department of Neurosurgery, Peking Union Medical College Hospital, Chinese Academy of Medical Sciences and Peking Union Medical College, Beijing 100730, China; liuph14@hotmail.com (P.L.); garywu12345@163.com (J.W.); mawb2001@hotmail.com (W.M.); 3 National Cancer Center/National Clinical Research Center for Cancer/Cancer Hospital, Department of Head and Neck Surgery, Chinese Academy of Medical Sciences and Peking Union Medical College, Beijing 100021, China; kongziren@pumc.edu.cn

**Keywords:** glioblastoma, fibroblast activation protein, tumorigenesis, immunosuppression, imaging biomarker, therapeutic target

## Abstract

Currently, glioblastoma (GBM) is the most common malignant tumor of the central nervous system in adults. Fibroblast activation protein (FAP) is a member of the dipeptidyl peptidase family, which has catalytic activity and is engaged in protein recruitment and scaffolds. Recent studies have found that FAP expression in different types of cells within the GBM microenvironment is typically upregulated compared with that in lower grade glioma and is most pronounced in the mesenchymal subtype of GBM. As a marker of cancer-associated fibroblasts (CAFs) with tumorigenic activity, FAP has been proven to promote tumor growth and invasion via hydrolysis of molecules such as brevican in the extracellular matrix and targeting of downstream pathways and substrates, such as fibroblast growth factor 21 (FGF21). In addition, based on its ability to suppress antitumor immunity in GBM and induce temozolomide resistance, FAP may be a potential target for immunotherapy and reversing temozolomide resistance; however, current studies on therapies targeting FAP are still limited. In this review, we summarized recent progress in FAP expression profiling and the understanding of the biological function of FAP in GBM and raised the possibility of FAP as an imaging biomarker and therapeutic target.

## 1. Introduction

As the most common malignant tumor of the central nervous system in adults [1], glioblastoma (GBM) is highly heterogeneous and invasive, contributing to the poor prognosis of patients. The standard therapy for newly diagnosed GBM includes maximum gross total resection, followed by radiotherapy plus concomitant and adjuvant temozolomide (TMZ). Although progress in therapy improves the patient survival time, the median overall survival remains at approximately only 14–16 months in randomized controlled trials [2], and the 5 year survival rate is approximately 6.8% according to the Central Brain Tumor Registry of the United States (CBTRUS) statistical report [1]. In addition, almost all GBM patients relapse, and recurrent GBMs progress more rapidly without well-defined standards of care currently [3]. The initial results of multiple phase III clinical trials, including immunotherapy, have been disappointing, and the complex immunosuppressive microenvironment is considered one of the main challenges imposed for immunotherapy in GBM [4]. Chemoresistance is a hallmark of recurrent GBM and one of the main challenges in the treatment of recurrent GBM [5]. Therefore, a solution to overcome chemoresistance and modulate the immunosuppressive microenvironment may be essential to improve therapeutic efficacy in GBM.

Fibroblast activation protein (FAP) is a member of the dipeptidyl peptidase family; it has catalytic activity when localized on the cell membrane or present in soluble forms, and it can act as a dipeptidyl peptidase or endopeptidase, preferentially cleaving postproline peptide bonds [6]. FAP monomers are inactive until they form active homodimers as well as heterodimers with cell membrane proteins such as integrins and urokinase-type plasminogen activator receptor (uPAR) [7]. In various malignant tumors, FAP is overexpressed and has been demonstrated to participate in tumor growth and progression; therefore, efforts in the clinical translation of FAP have been made, including imaging biomarkers, prognostic value and FAP-targeted therapies [8]. Recent studies discovered upregulated FAP expression within the GBM microenvironment [9] and associated FAP expression with tumorigenesis, immunosuppression and chemoresistance in GBM [10,11,12]. As an identifying surface marker of cancer-associated fibroblasts (CAFs) in other solid tumors, FAP also assists CAFs in suppressing antitumor immunity, promoting tumor growth and driving epithelial–mesenchymal transition (EMT) [13,14,15]. Researchers have identified distinct FAP/PDGFRβ dual-positive tumor-associated pericytes in the GBM microenvironment; these cells were demonstrated to be the major FAP-positive cells in GBM and might be CAF-like cells with tumorigenic roles in the GBM microenvironment [10]. However, despite growing research on the basic biology and functional roles of FAP in various cancers, comprehensive summaries of FAP in GBM are limited. Therefore, we summarized the recent progress in FAP expression profiling and in the understanding of the biological processes in GBM and discussed the potential of FAP as an imaging and prognostic biomarker and therapeutic target for remodeling the immunosuppressive microenvironment and reverting TMZ resistance.

## 2. FAP Is Expressed in Various Cell Types within the GBM Microenvironment

Except for in multipotent bone marrow stromal cells, α cells of Langerhans islands and some dermal fibroblasts surrounding hair follicles, the level of FAP expression remains low in most healthy tissue and cells [8]. It has been well established that FAP is prominently expressed in fibroblastic stromal cells in actively remodeling tissues, including tissues with chronic inflammation and fibrosis [16]; however, FAP expression is gradually being discovered in various additional cell types in the context of tumors.

In GBM, increased FAP expression has been detected mainly in mesenchymal stromal cells, astrocytes, glioma neural stem cells and sporadic CD45-positive cells [9,12]. These scattered CD45-positive cells might be fibrocytes or possibly a subset of macrophages originating in the bone marrow [8]. In murine lung cancer models, Arnold et al. characterized FAP+CD45+ cells as a subgroup of M2 macrophages [17]. Their findings are consistent with past studies in which FAP expression was discovered on macrophages in human breast cancer [18]. Additionally, Busek et al. discovered elevated expression of FAP at the protein level within the GBM microenvironment and frequently localized around dysplastic blood vessels, particularly in GBM of the mesenchymal subtype [9]. Ebert et al. later validated that blood vessels within GBM were highlighted by FAP expression, whereas normal blood vessels and cultured endothelial cells lacked FAP expression [12]. They further demonstrated that vessel-localized FAP expression was present on both endothelial cells and pericytes. Therefore, due to FAP expression on various cell types, including GBM cells, stromal cells, endothelial cells and pericytes, within the GBM microenvironment, FAP-targeted therapy could be extended to GBM, where FAP expression is limited to tumor cells, as well as their supporting vascular networks and stromal cells within the microenvironment, which play important roles in tumor progression.

CAFs are activated fibroblasts within the tumor microenvironment in multiple cancers, including breast cancer, pancreatic cancer and lung cancer, and are significantly involved in tumor progression and chemoresistance [19,20]. Although there is still limited evidence of CAFs or CAF-like cells in GBM [21], Li et al. recently identified increased FAP+/PDGFRβ+ cell populations in clinical glioma specimens as well as murine GBM models, suggesting that these cells are the major tumor-associated pericyte-like stromal cells within the GBM microenvironment [10]. They further discovered that these FAP+/PDGFRβ+ cells participated in TGFβ secretion in GBM, which is consistent with previous studies showing that GBM-activated pericytes secrete high levels of immunosuppressive cytokines, including IL-10 and TGFβ, and promote tumor growth [22,23]. These findings indicated that FAP/PDGFRβ dual-positive pericytes are a distinct CAF-like cell type in the GBM microenvironment with high expression of FAP and may play a significant role in tumor progression.

**Figure 1:**
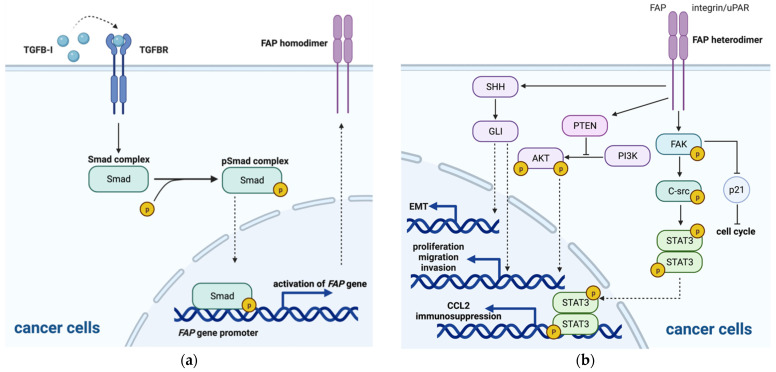
The signaling pathway in FAP regulation. (**a**) Regulation of FAP expression via the TGFβ signaling pathway in GBM cells; (**b**) downstream signaling pathway regulated by activated FAP heterodimers leading to various effects on cancer cells, including proliferation and invasion, immunosuppression and epithelial-mesenchymal transition (EMT).

Recently, a possible regulatory mechanism of increased expression of FAP in GBM was demonstrated. Rohrich et al. observed no specific FAP binding in U87MG cells in vitro; however, they discovered that FAP-specific radiotracer accumulated in U87MG tumor xenografts in vivo, and FAP immunohistochemistry showed various cells expressing FAP within the xenograft tumor, including tumor cells [24]. Their results demonstrated that FAP-negative GBM neoplastic cells might initially transform into FAP-positive cells after exposure to in vivo conditions, indicating that upregulation of FAP is possibly induced by crosstalk with environmental cells. Krepela et al. reported that transcripts encoding both FAP and TGFβ were upregulated in human GBM specimens, displaying a significantly positive correlation, and that TGFβ was found to induce FAP expression at the protein level and upregulate FAP activity in established human glioma cell lines (U87, U251 and U118), human brain vascular pericytes, glioblastoma-derived FAP+ mesenchymal cell cultures and glioblastoma-derived endothelial cell cultures, while no changes in FAP expression or enzymatic activity were observed in TGFβ-treated glioma stem-like cultures and human umbilical vein endothelial cells [25]. Their study further provided evidence that TGFβ mediates upregulation of FAP expression in U87 glioma cells through the canonical Smad-dependent TGFβ signaling pathway, in which activated TGFβ receptor induces phosphorylation of Smad (pSmad) and pSmad further directly activates transcription of the *FAP* gene by binding to its promoter (Figure 1a). In addition to the FAP+ pericytes discussed above, GBM cells, microglia and astrocytes have all been reported to secrete TGFβ [26,27]. In addition, a previous study discovered that TWIST1 was also able to bind to the *FAP* promoter and promote mesenchymal changes and cell invasion through FAP upregulation in SNB19 and/or T98G GBM cell lines [28]. All these findings indicate that FAP expression in GBM cells as well as several other cell types within the GBM microenvironment may be upregulated through autocrine or paracrine TGFβ signaling and mesenchymal transcription factors such as TWIST1. On the other hand, the mechanism by which low baseline FAP levels are maintained and unaffected by TGFβ-mediated upregulation of FAP expression in healthy tissues remains unclear, and further studies are warranted.

## 3. FAP Plays a Protumorigenic Role in GBM and Other Solid Tumors

Since current studies on the enzymatic and nonenzymatic activity of FAP in GBM are still limited, we reviewed the advances in FAP activity in other solid tumors, suggesting possible exploration directions for FAP in GBM. In addition, we also discussed the research progress on the functional roles of FAP in GBM.

### 3.1. Potential Substrates and Enzymatic Activity of FAP

Due to its dipeptidyl peptidase and endopeptidase activity, FAP is able to act on various substrates by forming active homodimers. Several substrates cleaved by FAP have been investigated and discovered recently, including collagen I and III, fibroblast growth factor 21 (FGF21) and neuropeptide Y (NPY) [29].

Previous studies have demonstrated that collagen I and III are cleaved by the soluble form of FAP in vitro [30,31]; however, recent studies have revealed the significance of collagen I cleavage. In an FAP-deficient murine model, the accumulation of intermediate-sized fragments was observed, and Fan et al. demonstrated that FAP mediated the ordered proteolytic processing of matrix metalloproteinase (MMP)-derived collagen cleavage products [32], indicating that FAP may play an important role in extracellular matrix modification. Additionally, a previous study demonstrated that FAP+ tumor-associated macrophages (TAMs) reside in human mammary adenocarcinoma [18], and Muliaditan et al. recently discovered that a collagen I-rich wound-like microenvironment helps maintain the FAP+ TAM phenotype in 4T1 mammary adenocarcinoma cell lines [33], which indicates that as the substrate of FAP, collagen I may also participate in educating infiltrated immune cells with upregulated FAP expression to promote tumor growth and invasion. Congruously, another study also discovered FAP expression on M2 macrophages in a transplanted model of pancreatic ductal adenocarcinoma, promoting tumoral immune suppression [17]; thus, it is inferred that collagen I helps maintain the M2 phenotype of macrophage infiltration in the tumor microenvironment with high expression of FAP, and the M2 phenotype is the anti-inflammatory phenotype of macrophages that suppresses immunity and enhances tumor proliferation [34]. In GBM, a recent study discovered that the invasiveness of GBM was associated with the proximity of fibrinogen to MMP-2 and MMP-9 [35], which suggests that the proteolytic process through MMP may also play an important role in GBM, and more studies are still needed to support the function of FAP and its substrates in GBM.

Another substrate, FGF21, is a key factor in metabolic regulation, and in response to various metabolic and cellular stresses, it is significantly upregulated to reduce excessive lipids and glucose and ameliorate tissue damage caused by metaflammation, which is a risk factor for tumor growth [36]. Recent studies have provided evidence that FAP is responsible for FGF21 cleavage and inactivation, in which selective chemical inhibitors, immune depletion or genetic deletion of Fap all stabilize recombinant human FGF21 in serum [37,38]; therefore, FAP may play an important role in metabolic regulation and tumor progression by rendering the FGF21 protein inactive.

Although DDP IV is the family member most similar to FAP, sharing approximately 50% homology, the substrates of FAP actually differ greatly from those of DDP IV. Utilizing in vitro assays in which potential substrates were added to soluble FAP-containing plasma, Wong et al. validated NPY, a substrate of DDP IV, as a substrate of FAP, while other DDP IV substrates, including substance P, peptide YY and B-type natriuretic peptide (BNP), were more efficiently cleaved by other proteases in human plasma than by FAP [39]. The NPY system modulates the immune microenvironment with various effects in different tumors [40]. In neuroblastoma, a high level of NPY is linked to poor prognosis, and multiple studies have demonstrated that NPY can promote tumor growth in an autocrine manner and induce vascularization through the NPY-mediated Y2 and Y5 receptor signaling pathways [41]. In contrast, Ruscica et al. discovered that a Y1 receptor antagonist reversed NPY-mediated tumor growth inhibition in prostate cancer [42], indicating that the NPY/Y1 receptor axis may contribute to tumor growth suppression; thus, FAP may modulate the immune microenvironment and have different effects on tumor growth through the NPY system in various cancers. More efforts have been made to identify novel substrates of FAP to clarify more detailed functions of FAP in cancers. Recently, several studies utilizing unbiased proteomic approaches and peptide library screens have uncovered several newly identified substrates, including chemokine CXCL-5 and IL-6, extracellular matrix protein fibrillin-2 and extracellular matrix protein 1 [43,44], although the direct activity of FAP in the cleavage process of each of these substrates needs further validation.

In conclusion, several substrates of FAP have been identified, including collagen I and III, FGFR21 and NPY, and the functional roles of FAP enzymatic activity have also been investigated. Via enzymatic cleavage of its substrates, FAP plays an important role in extracellular matrix modulation, infiltrated macrophage education, metabolic regulation and tumor promotion; therefore, inhibiting the enzymatic activity of FAP may be a potential strategy of antitumor therapy. Additionally, in GBM, several of the mentioned substrates were investigated, and possible functional roles of FAP in GBM were also proposed. We will discuss the current knowledge of FAP roles in GBM in a later context.

### 3.2. Signaling Pathways and Nonenzymatic Activity of FAP

Several downstream signaling pathways were found to be regulated by activated FAP heterodimers with α3β1 integrin or uPAR [45,46], leading to different functional effects on FAP-expressing cells and the microenvironment. These involved signaling pathways include the PI3K/AKT, RAS/ERK, sonic hedgehog (SHH), and focal adhesion kinase (FAK) pathways and other targets (Figure 1b).

#### 3.2.1. PI3K/AKT Signaling Pathway

In previous studies, the PI3K/AKT pathway was reported to induce pGSK-3β expression, therefore leading to cMyc-mediated cell cycle promotion, including cell proliferation [47], EMT through increased expression of the Snail and Slug transcription factors [48,49] and upregulated secretion of MMP2 and MMP9, promoting cell invasion [50]. PTEN, a known suppressor of the PI3K/AKT signaling pathway, was discovered to be downregulated by FAP in oral squamous cell carcinoma (OSCC) cells, and knockdown of FAP inhibited the growth and metastasis of OSCC cells in vitro and in vivo; therefore, it is suggested that FAP-mediated inhibition of PTEN activates the PI3K/AKT/GSK-3β pathway and that FAP acts as an oncogene [51]. Additionally, Wong et al. discovered that in OSCC cells, FAP mediates the ERK/RAS pathway to promote tumor progression and invasion [51]; however, Jia et al. found that the addition of PI3K or ERK inhibitors failed to restore the inhibitory effect of FAP in breast cancer cell lines, suggesting that the PI3K or ERK signaling pathway may not be a downstream target of FAP [52]. The findings above indicate that the PI3K/AKT and RAS/ERK signaling pathways may participate in FAP downstream regulation to promote tumor cell proliferation, migration and invasion, but the downstream regulation may differ in various cancers.

In GBM, the PI3K/AKT pathway is frequently hyperactive and was discovered to promote cell proliferation and invasion [53], and PTEN was reported to lose function [54]. According to a study by Verhaak et al., TCGA identified genetic deletions in only 85% of GBM, and approximately half of the remaining 15% did not show genetic alterations but displayed reduced expression of PTEN mRNA [55]. These findings suggest that there may be another diverse mechanism to downregulate PTEN expression in the remaining GBM. Therefore, the function of FAP in PTEN downregulation might explain the diversity in GBM; however, more investigations are needed.

#### 3.2.2. SHH/GLI Signaling Pathway

In addition to its effects via the PI3K/AKT pathway, Jia et al. further discovered that the SHH-FAP axis promoted cell growth, adhesion and migration potentially via the SHH/GLI signaling pathway in lung squamous cell cancer (SCC) cell lines, since only inhibitors of PI3K and SHH suppressed the increased motility of FAP-expressing cancer cells [56]. Another study in lung SCC specimens by Yue et al. demonstrated the reverse association between GLI expression and EMT markers, E-cadherin and β-catenin, and stimulation of the SHH/GLI pathway increased migration and suppressed E-cadherin expression in multiple lung SCC cell lines [57]. Considering that SHH is activated by FAP overexpression in A549 adenocarcinoma and SK-MES-1 SCC lung cancer lines [56], it can be inferred that FAP might play an indirect role in the EMT process through SHH/GLI regulation. The findings above support that the SHH/GLI signaling pathway enables FAP to be involved in tumor growth and migration and the EMT process.

Aberrant activation of the SHH/GLI signaling pathway was also discovered in GBM and was reported to be mediated by truncated GLI 1 (tGLI1) with gain of function, playing a central role in glioma pathogenesis and tumor progression [58]. Additionally, tGLI 1 was reported to regulate not only known GLI1 target genes but also other genes, including CD24, CD44 and VEGF; therefore, hyperactivation of the SHH/GLI signaling pathway in GBM also promotes cell growth, invasion and angiogenesis. GBM cell line analysis revealed that there was no deletion at the genomic level [59], suggesting that posttranscriptional splicing might be involved, which is not clearly understood. Thus, more investigation of FAP on *GLI* expression might be warranted to clarify whether FAP is a key factor in GLI truncation.

#### 3.2.3. FAK Signaling Pathway

FAK is a key mediator of signal transduction from cell membrane receptors, including integrin complexes and uPAR [45,60]. In a study utilizing ovarian cancer cell lines, Jia et al. observed decreased cancer cell proliferation and motility after the addition of FAK inhibitors [52], suggesting that FAK may be involved in tumor growth and migration promotion. Another study discovered that genetic deletion and inhibition of FAP elevated p21 in both an endogenous murine model of lung cancer and a xenograft murine model of colon cancer, and this elevation of p21 possibly occurred via the FAK signaling pathways [61]. p21 is well known to arrest the cell cycle; therefore, FAP is able to promote cell proliferation through suppression of p21 expression via the FAK and ERK signaling pathways. In addition to participating in tumor growth and migration, FAP has also been reported to promote immunosuppression via STAT3-CCL2 signaling. Inducing FAP expression in normal fibroblasts transforms them into cells with a CAF-like inflammatory phenotype with STAT3 activation and CCL2 upregulation, and this transformation is responsible for the formation of an immunosuppressive microenvironment. Yang et al. further identified that FAP induces STAT3 activation through the uPAR-dependent FAK/C-src/JAK signaling pathway [62]; thus, FAP promotes cell proliferation and immune suppression through the FAK signaling pathway.

In GBM, FAK has been reported to be activated by several upregulated cell surface receptors on GBM cells, including integrins and epidermal growth factor receptor (EGFR) [63]. Additionally, activation of the JAK/STAT signaling pathway was discovered to be prognostic in GBM [64]. Considering that overexpression of integrins in GBM cells and FAP can form heterodimers with integrins, the function of FAP in the FAK signaling pathway may be worth investigation.

### 3.3. Functional Roles of FAP in GBM

#### 3.3.1. Tumor Proliferation and Invasion

In multiple cancers, FAP has been proven to play an important role in tumor growth via promotion of tumor proliferation and invasion, and two hypotheses regarding FAP regulation have been proposed. On the one hand, it has been demonstrated that FAP modulates the extracellular matrix, therefore indirectly contributing to increased cell proliferation and migration. On the other hand, FAP can directly regulate proliferation and invasion through intracellular signaling pathways and transcriptional regulation of genes associated with the cell cycle. In GBM, migration and invasion are considered to be facilitated by protease expression through digestion of the extracellular matrix [65]. Brevican is a proteoglycan that is enriched in human brain tissues and is reported to be cleaved by several secreted MMPs to promote the invasion of glioma cells [66]. As discussed above, FAP is responsible for the cleavage of MMP-cleaved collagen products; therefore, it could be inferred that FAP may participate in brevican cleavage. Mentlein et al. further discovered that FAP-silenced GBM cells migrated similarly through noncoated or basal laminal-coated membranes but much more slowly through membranes coated with brevican [67], validating the functional role of FAP in promoting GBM invasion through digestion of brevican. Furthermore, FAP was found to be coexpressed with CD90 on tumor cells [12], which has been demonstrated to drive GBM cell invasion [68], suggesting that FAP might promote cell invasion via functional interactions with CD90. Nevertheless, the direct role of FAP expression on tumor cells in the regulation of cell proliferation through intracellular signaling pathways remains to be investigated, and studies on the enzymatic role of FAP on cells other than tumor cells within the GBM microenvironment remain to be addressed.

#### 3.3.2. Angiogenesis

An initial study observed vessel-localized FAP expression in GBM; however, the identity of those cells remained unclear [9]. A recent study revealed that these cells might be a mix of vascular pericytes, endothelial cells and glioma stem cells [12]. The researchers discovered that some of the perivascular FAP+ cells more resembled tumor cells than pericytes; therefore, they were assumed to be glioma stem cells, which have been reported to preferentially reside in the perivascular niche [69]. Furthermore, the researchers demonstrated that glioma stem cells could differentiate into tumor pericytes, and analysis of the Ivy GBM dataset showed intense FAP expression in areas of microvascular proliferation, suggesting the angiogenetic role of FAP expression on glioma stem cells in GBM. Current knowledge about the angiogenic function of FAP in GBM remains limited, and more investigation is warranted to validate the possibilities.

#### 3.3.3. Immunosuppression in the Tumor Microenvironment

As we have discussed above, FAP+ pericytes have been identified as the major CAF-like cells in the GBM microenvironment and are actively involved in the secretion of TGFβ, which has been identified as an immunosuppressive cytokine [10]. Therefore, the acquisition of immunosuppressive properties by FAP+ pericytes contributes to the depletion of antitumor immunity in GBM, and thus, these FAP+ pericytes seem to have immunosuppressive roles similar to those of CAFs in other solid cancers [70,71,72]. In addition, FAP expression was discovered on CD45+ cells in GBM, and these cells might represent a subgroup of M2 macrophages [8,17], as they display protumorigenic functions and suppress antitumor immunity. Furthermore, receptors Y1 and Y2 of NPY, which is the substrate of FAP, were discovered to be highly expressed in GBM cells. Y2 receptor agonists specifically stimulated GTPγS binding, and nanomicelles targeting the Y1 receptor prolonged the survival rate in GBM murine models, indicating that NPY-activated Y1 and Y2 receptors might participate in tumor growth [73,74]. Previous studies have identified the NPY system as having a functional role in the modulation of the immune system, promoting tumor growth in various cancers [40], indicating that FAP may play a potential modulatory role in the immune microenvironment via activation of the NPY system; however, direct evidence of FAP activity is still needed.

#### 3.3.4. EMT and TMZ Resistance

EMT is defined as a transition from an epithelial to a mesenchymal phenotype, which enables elevated invasion and migration of tumor cells. Multiple previous studies have suggested that TMZ resistance in GBM is related to EMT promotion, which is accomplished through the upregulation of MMPs and downregulation of E-cadherin mediated by the TGFβ and PI3K/AKT signaling pathways [75,76]. As discussed above, these signaling pathways are potentially downstream targets of FAP regulation, suggesting a possible role of FAP in EMT and TMZ resistance. A recent study observed miR-204 downregulation in U251MG-resistant cells, discovered that miR-204 expression was negatively associated with FAP levels in human GBM tissues and revealed that miR-204 might reverse TMZ resistance [77]. Yang et al. further concluded that miR-204 possibly reversed TMZ resistance and inhibited cancer-initiating cell phenotypes by degrading FAP in U251MG-resistant cells, indicating a potential role of FAP in TMZ resistance in GBM. In other words, FAP may play a functional role in promoting TMZ resistance in GBM by promoting the EMT process, but more direct evidence is needed to validate this hypothesis.

## 4. Future Clinical Applications of FAP

### 4.1. FAP as a Potential Imaging Biomarker

**Figure 2:**
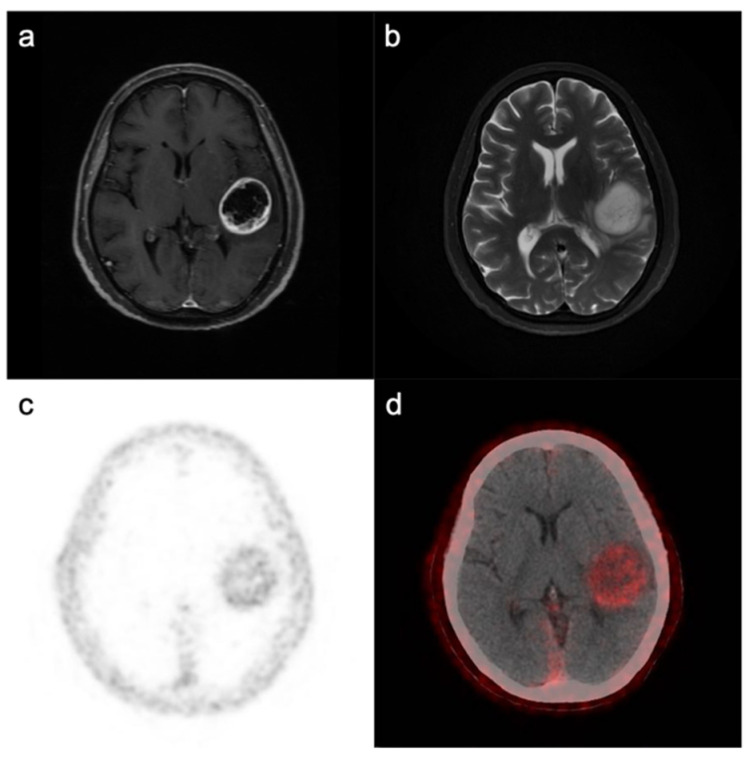
Magnetic resonance imaging (MRI) and FAPI PET/CT in GBM. An IDH-wildtype GBM from a 71-year-old woman displayed ring-like contrast enhancement with hypointense T1-weighted and hyperintense T2-weighted signals (**a**,**b**) from central tissue. ^68^Ga-FAPI PET and merged PET/CT images (**c**,**d**) showed elevated radioactivity in the whole tumor area (including ring-like contrast enhancement and noncontrast-enhanced tissue).

Due to the unique overexpression and membrane localization of FAP on various cells within the GBM microenvironment, FAP is considered a potential molecular diagnostic biomarker, and FAP inhibitors (FAPIs) have been under investigation for PET imaging in several cancers [78], broadening the applications for noninvasive tumor diagnosis, grading stratification and planning for surgery and radiotherapy. Both biodistribution studies in U87MG tumor-bearing murine models and small animal PET studies have shown increased immuno-PET radiotracer retention in tumors over time, and the accumulation of radiotracers was a specific marker of FAP expression, indicating that FAP might be a potential imaging biomarker for GBM diagnosis [11]. Recently, the first in-human clinical pilot study utilizing ^68^Ga-FAPI PET further observed increased tracer uptake in IDH-wildtype and IDH-mutant GBM and WHO grade III IDH-mutant astrocytoma but not in WHO grade II astrocytoma [24]. Furthermore, they validated that FAP-positive cells existed in GBM and WHO III astrocytoma specimens by immunohistochemistry. In our own clinical practice, GBM showed high uptake and image contrast on ^68^Ga-FAPI PET/CT (Figure 2). These findings raise the possibility that FAP might be used as an imaging biomarker allowing noninvasive determination of tumor malignancy. Their study also showed tracer uptake in GBM with intratumoral heterogeneity [79], and previous studies have shown that a high level of FAP expression correlates with increased aggressiveness of GBM with increased invasiveness and EMT [9,67]. Therefore, more studies assessing the potential of FAP as an independent imaging biomarker for delineating the most malignant parts within GBM for more precise biopsy and planning of surgery and radiation therapy are warranted to open up new applications. However, it is also important to note that FAP is upregulated in chronic inflammation and fibrosis; therefore, distinguishing GBM from other inflammatory or autoimmune neurological diseases needs to rely on the clinical manifestations and characteristics of multimodal imaging.

On the other hand, FAP signaling showed a moderate positive correlation with relative cerebral blood volume (rCBV) but no correlation with apparent diffusion coefficient (ADC), suggesting that FAP is an independent marker of local perfusion or cell density [79]; therefore, Rohrich et al. considered that FAP might similarly deliver complementary, independent information in distinguishing progression and pseudoprogression, such as rCBV and ADC, which can improve the diagnostic accuracy in this situation. However, FAP has been reported to be upregulated under multiple reactive conditions, including postinflammation after radiation therapy [16]. It remains unclear whether FAP can be utilized to discriminate progression from pseudoprogression after radiation therapy, and more investigation is needed.

### 4.2. Prognostic Value of FAP

Additionally, increasing evidence shows the possible prognostic value of FAP in several malignant solid tumors [8]. Recent studies have associated a high level of FAP expression with high-grade glioma [9,10,24]. Based on data analysis of glioma TCGA (*n* = 667) and CGGA datasets (*n* = 633), the mRNA levels of FAP were significantly elevated in GBM compared to healthy brain tissues and lower grade glioma tissues, and a higher level of FAP was related to poorer prognosis across gliomas of all grades (TCGA dataset analysis HR = 0.36; CGGA dataset analysis HR = 0.51) [10]. However, another study conducted survival analysis with both a GBM patient cohort (*n* = 42, *p* = 0.10) and two publicly available datasets (*n* = 155, *p* = 0.69; *n* = 372, *p* = 0.30), and no relationship between FAP expression and survival in GBM patients was observed [9]. Since FAP is typically overexpressed in higher grade glioma, especially GBM [24], the previously observed negative correlation of the FAP expression level with survival may have been caused by the large proportion of GBM cases with high FAP expression in the analysis group. On the other hand, FAP expression was prominent in the mesenchymal subtype of GBM [9], which renders a more aggressive subtype of GBM with a worse prognosis [80]. Therefore, the prognostic value of FAP in GBM remains controversial and may not be valid, and more evidence is needed.

### 4.3. Advances in FAP-Targeted Therapy

Since the FAP expression level is reported to be low in healthy tissues and elevated in multiple cancers, various efforts to utilize FAP as a therapeutic target have been made. Talabostat (Val-Boro-pro, PT-100, BXCL-701) is designed to inhibit the enzymatic activity of FAP, and several phase I and II clinical trials have shown promising results in multiple cancers, including pediatric solid tumors, metastatic colorectal cancer, melanoma and nonsmall-cell lung cancer, with improvements in response rates and prolonged states of stable disease. Nevertheless, several side effects were also noted, and most of them were caused by cytokine storms [81,82,83]. FAP vaccination is another therapeutic strategy and has also been investigated utilizing DNA vaccines directly targeting FAP, FAP-expressing whole cell vaccines or dendritic cell vaccines with FAP coexpression [84]. FAP vaccination has been attempted in several studies, in which tumor growth and metastasis were suppressed and survival time was prolonged, and there was increased infiltration of CD8+ T cells in a murine model inoculated with colon or breast cancer cells [85,86]. Chimeric antigen receptor (CAR) T cell therapy is one of the most popular immunotherapies, and FAP has been investigated as a target for cancer cell recognition. Several preclinical studies observed successful depletion of FAP-expressing cells and overall survival improvement in murine models [87,88]. However, one study utilizing CAR-T cell therapy failed to demonstrate efficacy and showed severe bone toxicity and cachexia [89]. Thus, more studies are still needed to optimize the design and dose of CAR-T cell therapy targeting FAP.

In GBM, FAP expression was found in both tumor cells and stromal components, including mesenchymal cells and pericytes surrounding the vascular network; therefore, FAP is considered to be a potential therapeutic target for strategies aiming to destroy tumor cells, their supporting vascular networks and extracellular matrix integrity. Several preclinical studies have demonstrated the possibility of FAP as a therapeutic target. One ongoing study recently released data suggesting promising efficacy for FAP-targeting CAR-T cell therapy in a mouse xenograft model of GBM and a lack of toxicity [12]. A recent study reported data from a murine GBM model for an oncolytic adenovirus that infects and depletes FAP+ pericytes and GBM cells, highlighting the potential utility of oncolytic virus immunotherapy targeting FAP in GBM [10]. However, further research is warranted to validate this new strategy and especially to demonstrate whether viruses targeting the GBM stroma can lead to a survival benefit. On the other hand, it is worth noting that although no FAP expression was detected in healthy brain tissue from nontumor-bearing patients [9], Krepela et al. found detectable FAP RNA but not protein in tumor-adjacent tissues of a glioma xenotransplantation mouse model [90]. These data suggest possible effects of glioma on FAP expression in distal tissues, and more consideration needs to be given to the use of FAP as a diagnostic biomarker and side effects of FAP as a therapeutic target in clinical practice. Currently, FAP research is still in the early preclinical stage, and more studies of FAP expression and its functional role are warranted to inform future clinical strategies utilizing FAP.

## 5. Discussion

In this review, we summarized the recent progress in FAP expression and functional roles within tumor microenvironments. Initially, FAP was reported to be prominently overexpressed in fibroblasts within the cancer microenvironment; however, recently, FAP expression was also discovered on tumor cells in several types of tumors [5]. The protumorigenic activity of FAP was accomplished through indirect enzymatic activity in the modulation of the extracellular matrix and a direct role in the transcriptional upregulation of related genes to promote tumor growth, tumoral vascularization and immune suppression. Several preclinical and clinical studies have shed light on the future of FAP-targeting therapy in multiple cancers.

Recently, upregulation of FAP expression in various cell types within the GBM microenvironment has been demonstrated, including GBM cells, stromal mesenchymal cells, pericytes and GBM-derived endothelial cells; however, more studies considering the detailed protumorigenic effects of FAP are warranted. Although our knowledge about the biological behavior of FAP in GBM remains incomplete, the potential of FAP as an imaging biomarker and therapeutic target has been displayed. FAPI, an FAP-specific small molecule with favorable pharmacokinetics in vivo and in vitro [32], has been broadly utilized in PET/CT and has shown promising results as a diagnostic biomarker with grading value in GBM. On the other hand, the future applications of FAP-specific PET also include biopsy/surgery radiation planning and distinguishing between progression and pseudoprogress after radiation, where more validation is warranted.

There are still several limitations of this review. Although FAP has been thoroughly investigated and discussed in other types of tumors, there are limited investigations on FAP in GBM, and molecular biology and biochemistry studies are especially lacking. In this review, we summarized the current understanding of FAP regulation and functional roles broadly in multiple other tumors and not restricted to GBM. However, those studies in other tumors may provide possible exploration directions in future investigations to understand the functional roles and mechanisms of FAP in GBM. Additionally, although upregulation of FAP was discovered at both the protein and mRNA levels, there was only a moderate correlation between FAP mRNA and protein expression in GBM [9]. This suggests that in addition to the TGFβ signaling pathway and mesenchymal transcription factors, FAP expression may also be regulated by posttranscriptional mechanisms, which might also explain why FAP expression can be maintained at a low level in healthy tissues despite TGFβ mediation. Further investigations on posttranscriptional mechanisms may be warranted.

Additionally, several problems remain to be solved in the clinical practice of FAP in GBM. As an imaging biomarker, FAP is also upregulated in actively remodeling tissues; therefore, the distinction between GBM and other neurological inflammatory or autoimmune diseases relies on clinical manifestations and multimodal imaging, including MRI. Studies on the practical relevance between FAP expression level and prognosis in GBM patients rather than glioma patients are contradictory, and more data are needed. There is limited evidence on the ability of FAP to identify the heterogeneity within GBM and discriminate between the invasion borders and adjacent brain tissues. Although a few preclinical studies have assessed the possibility of FAP as a therapeutic target in GBM, the design of FAP-targeted therapy in GBM is still lacking, and the main challenges for FAP-targeted therapy are the high heterogeneity of GBM, the effects of GBM cells on distant tumor-adjacent brain tissues to express FAP and the expression of FAP in other reactive conditions including fibrosis and inflammation. Therefore, finding a coexpression profile of FAP within the local GBM environment is essential to distinguish FAP expression within GBM and in other conditions. Additionally, more basic studies concerning the mechanism and functional roles of FAP in GBM are still needed to further support the clinical practice of FAP in GBM. In addition, more information is needed to understand the mechanism by which FAP reverses TMZ resistance in GBM to transform FAP in clinical practice to reverse chemoresistance.

In conclusion, current studies have discovered upregulated FAP expression in GBM and proposed potential roles of FAP in GBM which are not yet well characterized in GBM; however, preclinical studies have shown the potential of FAP in clinical practice, while more investigation is needed to fulfill our understanding of FAP functional roles and overcome the challenges we are facing to improve FAP targeted therapy in GBM.
